# Epidemiology of Needle Stick Injuries at a Tertiary Care Hospital in Trinidad From 2011 to 2016: A Retrospective Study

**DOI:** 10.7759/cureus.108832

**Published:** 2026-05-14

**Authors:** Rajeev P Nagassar, Stanley Giddings, Damion Basdeo, Keston Daniel, Sandyn Bassarath, Elise Renaud

**Affiliations:** 1 Microbiology, Sangre Grande Hospital, Sangre Grande, TTO; 2 Internal Medicine, University of the West Indies, San Fernando, TTO; 3 Internal Medicine, Sangre Grande Hospital, Sangre Grande, TTO; 4 Surveillance, Caribbean Public Health Agency, Port of Spain, TTO; 5 Engineering, University of the West Indies, Port of Spain, TTO; 6 Surgery, Sangre Grande Hospital, Sangre Grande, TTO

**Keywords:** blood borne pathogens, healthcare workers, infection control, needle stick injury, occupational exposure, post‑exposure prophylaxis, trinidad and tobago

## Abstract

Background: The most significant infections via needle-stick injuries (NSIs) involve hepatitis B, hepatitis C, and HIV, and the most common device is a hollow-bore needle or suture needle. This study aimed to determine the epidemiology and associated factors that contribute to occupational exposures to blood and bodily fluids at a hospital in South Trinidad and Tobago.

Methods: This study was conducted retrospectively from 2011 to 2016 in one tertiary public health institution in Trinidad and Tobago. Data were extracted from incident reports, occupational health records, and infection prevention and control (IPC) unit documentation. Primary outcomes included demographic characteristics, occupation, and departmental distribution of NSIs. Secondary outcomes assessed the circumstances of injury, anatomical site, glove use, laboratory investigations, and administration of post‑exposure prophylaxis (PEP).

Results: A total of 221 healthcare workers reported NSIs, with a mean age of 34.97 years. Most affected individuals were women and clinical staff. "Other" (e.g., psychiatry, or in transit outside wards/units) accounted for the highest proportions of injuries, followed by internal medicine, while the IPC unit received the majority of first reports. Procedures were the most common activity associated with injury, followed by discarding needles and recapping. Fingers were the most frequently affected sites, particularly the right index and left ring fingers. Documentation gaps were notable, especially regarding glove use and time to reporting. PEP was administered in most cases, and official reporting to the IPC unit was associated with a higher likelihood of receiving PEP. Laboratory testing commonly included renal and liver function tests, complete blood counts, and screening for blood‑borne pathogens.

Conclusion: NSIs reflect ongoing challenges in occupational safety. Strengthening IPC training, improving reporting practices, promoting safer sharps handling, and implementing safety‑engineered devices are essential to reducing NSI incidence and improving post‑exposure management.

## Introduction

A WHO report from 2003 notes that more than 3 million healthcare workers (HCWs) experience percutaneous exposure to infectious diseases annually [1]. Some of the most clinically significant infections are hepatitis B, hepatitis C, and HIV, and the most common device is a hollow-bore needle or suture needle. This report also mentions that 37% of hepatitis B, 39% of hepatitis C, and 4.4% of HIV cases among HCWs occur due to needle-stick injuries (NSIs) and contaminated needles in the 20-65-year age group [1].

Underreporting of NSIs is a frequent concern. Chadee et al. looked at inoculation injuries in hospitals in Trinidad and Tobago from 1991 to 1997, and they found that out of the 397 inoculation injuries over a seven-year period, 305 (76.8%) were due to NSIs. Nurses, followed by doctors and then ward maids, were the main recipients of inoculation injuries [2]. Over the past decade, infection prevention and control (IPC) efforts have developed across Trinidad and Tobago. IPC units have been established at each regional health authority, and a national IPC manual, surveillance, and training of HCWs have been developed [3]. New data on NSIs and associated factors are needed. This will help to evaluate methods to further prevent NSIs and guide the management of such occupational injuries [4]. Nagassar et al. performed a similar study at a tertiary institution in East Trinidad, which evaluated the factors involved in NSIs [5]. Important findings included that the majority of the NSIs occurred in the internal medicine department (33.3%), registered nurses (26.8%) were the most affected category of staff, the majority of incidents were reported to the accident and emergency department (89.1%), the majority of NSIs occurred on the fingers (52.9%), tests for infective blood-borne diseases (HIV, HBV, and HCV) were performed in most cases but not all (98.6%), more than half had post-exposure prophylaxis (PEP) administered (58.8%) and one person (1.3%) had seroconversion [5].

We thus utilised a methodology and formulated objectives similar to this study published in 2021 by Nagassar et al [5]. This study aimed to determine, retrospectively, the epidemiology and associated factors that contributed to occupational exposures to blood and bodily fluids at a hospital in South Trinidad and Tobago between 2011 and 2016.

## Materials and methods

Study centre and design

We conducted a retrospective cross-sectional study to identify all individuals who self-reported NSIs at a tertiary care hospital in Trinidad for the period 2011 to 2016. The data was collected from the IPC unit reports as well as from physician records at the infectious disease clinic. The study took place in San Fernando General Hospital, which is the main tertiary institution located in the South West Regional Health Authority (SWRHA). This is one of the five Regional Health Authorities (RHAs) in Trinidad and Tobago.

Inclusion and exclusion criteria

All individuals with self-reported NSIs from 2011 to 2016 were included. Duplicate entries for the same event were excluded. Self-reported NSIs that occurred in individuals under 18 years of age were also excluded.

Sample size

To calculate the sample size for a self-reported NSI study conducted retrospectively from 2011 to 2016 in one tertiary public health institution in Trinidad and Tobago, the approach differs slightly from a typical prospective cross-sectional study. For a retrospective descriptive design for our study, which spans 2011-2016, we analysed existing data (e.g., incident reports, occupational health records, or survey recollections of past events). In such retrospective descriptive studies, the sample size may be defined by data availability rather than prospective recruitment [6]. However, the formula used is as stated below [7]:

The sample size was calculated using standard formulas for estimating proportions, with adjustment for a finite population. First, an initial sample size (x) was calculated based on the desired confidence level and estimated population proportion:

$x = Z^2 p(1 - p)$

Where Z is the Z-score corresponding to the selected confidence level (e.g., 1.96 for 95% confidence), and p is the estimated proportion of the population with the characteristic of interest. 

Because the study population is finite, the sample size was adjusted using the finite population correction (FPC):

$n = \frac{N \times x}{((N - 1)E^2 + x)}$

Where n is the required sample size, N is the total population size, and E is the desired margin of error (expressed as a proportion, e.g., 0.05).

The margin of error associated with the final sample size was calculated as:

$E = Z \sqrt{\left[\left(\frac{p(1 - p)}{n}\right) \times \left(\frac{N - n}{N - 1}\right)\right]}$

This formulation incorporates both sampling variability and the finite population correction factor. Using a prevalence of 40% as estimated by Matsubara et al., a study published in 2017, incorporating a similar setting (the developing nation of Lao), the formula above yields a sample size of approximately 296 participants [8].

Outcomes

Primary Outcomes

The primary outcomes were the epidemiology of individuals involved in self-reported blood and bodily fluid exposures, including NSIs at SFGH in Trinidad during the period 2011 to 2016, and the departments in which the NSIs were first reported, as well as the departments in which the self-reported NSIs occurred during the same timeframe.

Secondary Outcomes

The secondary outcomes were the circumstances or procedures being performed when the self-reported NSI occurred at SFGH during the 2011 to 2016 period, and the site of injury. Additional secondary outcomes included whether gloves were worn at the time of the self-reported NSI, the blood tests performed after the injury, whether PEP medication was administered, and the estimated time to PEP administration following the self-reported NSI at SFGH during the same timeframe. The PEP referred to in this study was that given for HIV.

Data collection and analysis

A data collection tool was used to collect the relevant demographic data and needle stick injury data from the IPC units and infectious disease unit (see Appendix). To determine the epidemiology of needle stick and bodily fluid exposures, descriptive statistics were used. This was presented as frequency counts and percentages to summarise the data. Also, variables such as demographics (e.g., age and sex), the setting of exposure, and the type of exposure were assessed with descriptive analysis. Mean, median, and standard deviations were used for continuous variables, while categorical variables were expressed as proportions. Chi-squared analyses were conducted to identify significant associations between categorical variables and PEP. Logistic regression was performed to determine which combination of variables and interaction terms best explained the variation in PEP. Kaplan-Meier survival analyses were conducted on the two variables most significantly associated with PEP. Cox proportional hazard ratios were calculated to assess the impact of these variables on the timing of PEP administration, providing estimates of relative risk over time while adjusting for potential confounders. A p-value of <0.05 was considered significant, unless otherwise stated.

Management of incomplete and missing data

Given the retrospective design, missing and incomplete data were anticipated due to variability in documentation across IPC records and clinical notes. Data completeness was assessed for all variables, and missing values were classified as "not documented" where appropriate. A complete-case (available-case) approach was used for analysis; however, variables with substantial missingness were retained and reported as separate categories to preserve sample size and reflect real-world reporting practices. No imputation was performed due to the high likelihood of data being missing not at random (MNAR), particularly for variables dependent on reporting behaviour. The extent and pattern of missingness were explored descriptively, and associations between missingness and key categorical variables were assessed using the chi-square test to evaluate potential systematic bias. Sensitivity comparisons between complete and incomplete records were undertaken where feasible.

## Results

Demographic characteristics

The population of the study comprised 221 HCWs who had NSIs from 2011 to 2016 at SFGH. Out of these, the mean age was 34.97 ± 10.15 years. There were a total of 41 males and 180 females, mainly clinical staff/medical staff (n=106; 47.96%). See Table 1 for additional demographic details.

**Table 1:** Descriptive data for gender and general occupational category (n=221) Single hyphen "-" indicates an empty cell

Variables	%	N
Sex	-	-
Female	81.4	180
Male	18.6	41
Occupation	-	-
Medical/clinical staff	47.96	106
Student/intern	20.36	45
Other	31.67	70

Place where the injury occurred and where it was reported

Most of the injuries occurred in "other" (40.27%; n=89), closely followed by the internal medicine wards (39.82%; n=88). The highest number of injuries was first reported to the IPC Unit (46.61%; n=103). Table 2 includes further details on the location where the injury occurred and where it was first reported.

**Table 2:** Descriptive data showing where incident was first reported and where injury occurred Single hyphen "-" indicates an empty cell. * "Other" refers to injuries that did not occur in the predefined categories (e.g., psychiatry or in transit outside wards/units).

Variables	%	N
Where first reported		
Accident and emergency department	42.08	93
Health centres	8.14	18
IPC unit	46.61	103
Other	3.17	7
Where injury occurred	-	-
Other*	40.27	89
Medical ward (internal medicine)	39.82	88
Accident and emergency department	8.6	19
Surgical/urology/paediatrics/orthopaedics/ENT unit	6.79	15
Other units	4.52	10

Mechanism of injury: circumstance and location on the body

Most injuries occurred while performing a procedure, 37.11% (n=82), followed by discarding, 16.74% (n=37), and removing the needle or recapping, 12.67% (n=28). Also, the site most commonly injured on the hand was the left ring/fourth finger, 23.64% (n=52). The second most common site of injury category was "others", which involved all areas other than the hands or fingers, 17.27% (n=38). See Table 3 for circumstances and site of injury.

**Table 3:** Descriptive data showing the circumstance in which the injury occurred and location of injury to body Single hyphen "-" indicates an empty cell "Circumstances" data were missing for two participants. "Site-of-injury" data were unavailable for 13 participants.

Variables	%	N
Circumstances		
Other	33.48	72
Performing a procedure	37.11	82
Discarding	16.74	37
Removing or recapping	12.67	28
Missing data	0.9	2
Site of Injury		
Left thumb	0	0
Right thumb	4.09	9
Left little finger	5.91	13
Right little finger	1.36	3
Left index finger	0	0
Right index finger	15.91	35
Left ring/fourth finger	23.64	52
Right ring/fourth finger	1.36	3
Left middle finger	1.36	3
Right middle finger	11.82	26
Left hand	0.45	1
Right hand	6.36	14
Not documented	5	11
Other	17.27	38
Missing data	5.8	13

Reporting, administration, and the type of PEP given

The documentation of the time between the event and reporting occurred in 27.6% (n=61) of the cases. Of this, it was reported immediately in 11.76% (n=26) of the cases. But the majority did not have documentation of the time elapsed between the event and reporting, 72.4% (n=160). 

With regards to an official report being made to IPC, the majority were reported (61.99%; n=137), 35.29% (n=78) were not documented if reported, and 2.71% (n=6) were not reported. Using the chi-squared analysis, an official report filed to IPC was found to be a statistically significant determinant of whether PEP was administered (p value=0.00086) (Table 4). PEP was given in the majority of cases (94.1%; n=208) (Table 5). This mainly consisted of Combivir and Kaletra 82.81% (n=183). Combivir alone accounted for 4.98% (n=11), and other highly active antiretroviral therapy (HAART) accounted for 6.34% (n=14).

**Table 4:** Descriptive data showing whether the PEP was given and if documented PEP: post-exposure prophylaxis

PEP	%	N
Yes	94.1	208
No	5.4	12
Not documented	0.5	1

**Table 5:** Cross-tabulation and chi-squared analysis for official reports filed to IPC Single hyphen "-" indicates an empty cell Pearson chi-square value ≈ 14.12 PEP: post-exposure prophylaxis; IPC unit: infection prevention and control unit

Variable (Group)	PEP (N) (%)		p-value
Official report filed to IPC	Yes	No	
Yes	135 (64.90)	1 (9.09)	0.00086
Not documented	68 (32.69)	9 (81.82)	
No	5 (2.40)	1 (9.09)	

Wearing of gloves at the time of injury and blood testing

The majority of persons involved in NSIs did not have documentation on whether they were wearing gloves or not, 80.53% (n=178). However, of those documented, more persons were found to be wearing gloves, 14.93% (n=33), compared to those not wearing gloves or where glove use was not applicable (4.52%; n=10). The majority of persons underwent RFT, LFT, and CBC testing initially after getting injured (59.28%; n=131). The other most common tests included HIV, HepB, HepC, VDRL, accounting for 22.62% (n=50).

Variables and their influence on the time to PEP administration

**Figure 1:**
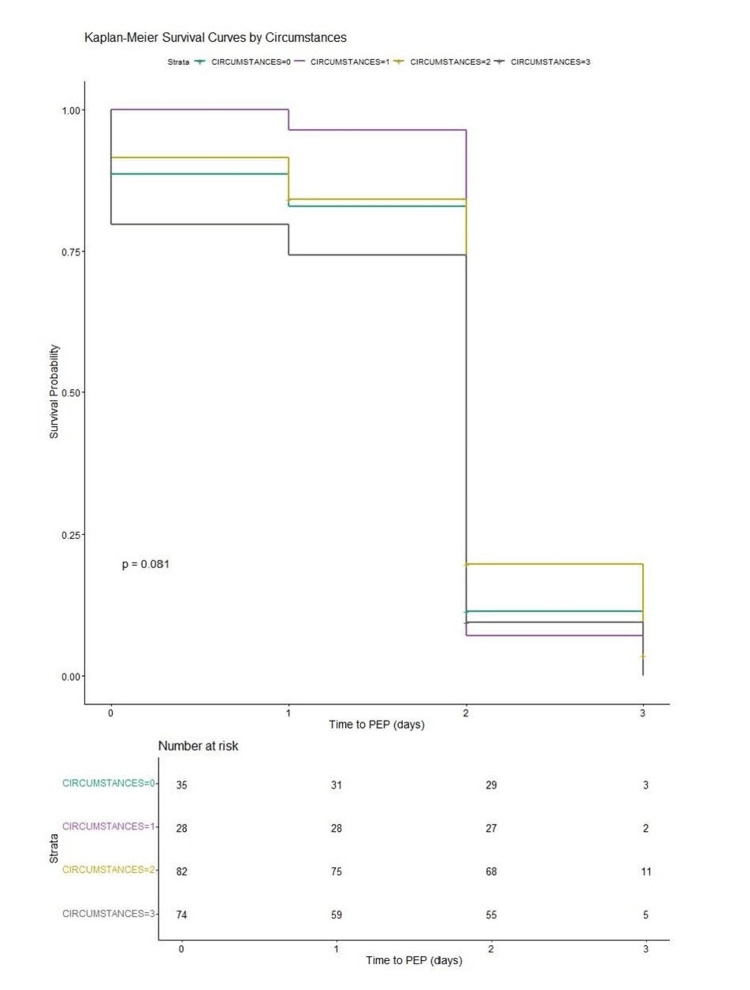
Kaplan-Meier survival curves by circumstance and the time to PEP (CI=0.95, p=0.081) The "strata" part (distinct subgroups) for Figure 1 are as follows: Circumstance=0 (discarding); circumstance=1 (removing or recapping); circumstance=2 (performing a procedure); circumstance=3 (e.g., handing to administer medication). PEP: post-exposure prophylaxis

The Kaplan-Meier curve is a step-function graph that shows the probability of survival or the time until an event occurs, e.g., receiving PEP. The Kaplan-Meier curve for the circumstance of injury versus time to receive PEP is shown in Figure 1. This curve indicates some correlation between performing a procedure (circumstance=2) and PEP, as the curve is seen as distinct from the other curves; however, it is not statistically significant. However, circumstances overall show some evidence of influence, though not conclusive (p = 0.081).

**Figure 2:**
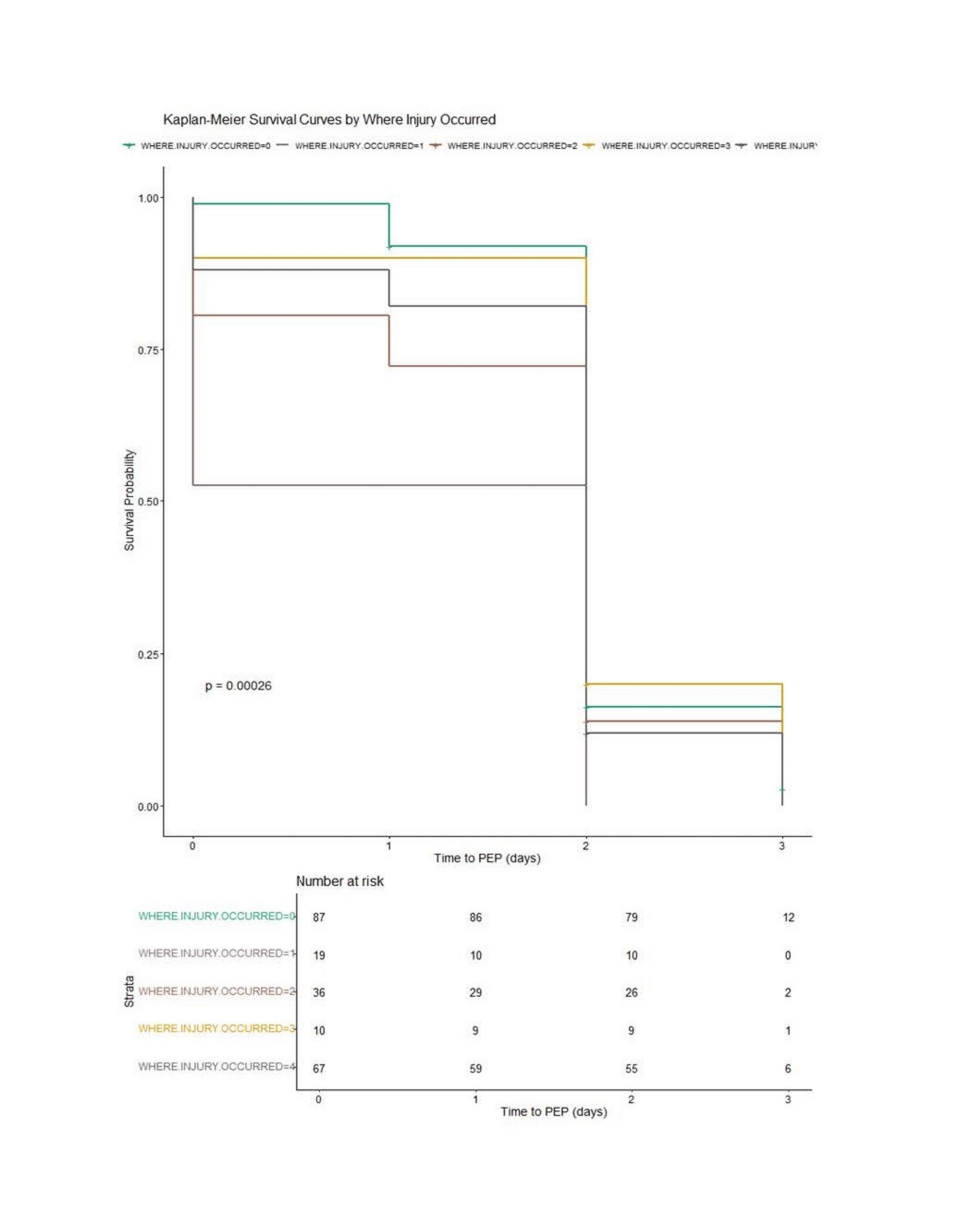
Kaplan-Meier survival curves by where the injury occurred and the time to PEP (CI=0.95, p=0.00026). This shows Group 0 (medical ward) having the highest survival probability and Group 3 (other units, e.g., psychiatry) the lowest. The "strata" part (distinct subgroups) for Figure 2 are as follows: Where injury occurred=0 (medical ward); where injury occurred=1 (accident and emergency); where injury occurred=2 (surgical/urology/paediatrics/orthopaedics/ENT Units); where injury occurred=3 (other units, e.g., psychiatry); where injury occurred=4 (others, e.g., unspecified ward or unit) PEP: post-exposure prophylaxis

Considering this, the Kaplan-Meier curve for "where the injury occurred" and receiving PEP indicated a higher chance of survival; there was evidence of association and overall statistical significance (See Figure 2). Therefore, the curves for Group 0 (medical ward) indicate the highest survival probability vs Group 3 (other units, e.g., psychiatry), with the lowest probability of receiving PEP after injury.

In conclusion, for the survival analyses, the variables that showed impact on survival after PEP administration were "circumstances" (Figure 1) (CI = 0.95, p = 0.081) and "where injury occurred" (Figure 2) (CI = 0.95, p = 0.00026). However, only "where the injury occurred" was statistically significant for improved survival. Additionally, multivariate logistic regression was performed to correlate and assess predictors influencing PEP administration. The main effect-only model was done for the "test done" but was not significantly correlated (OR=1.67, p = 0.13). Multivariate logistic regressions were done to assess predictors of PEP administration with "where the injury occurred" and "test done," but these were not significant predictors (OR = 1.95, p = 0.13). Similarly, the interaction between "where injury occurred' and "wearing gloves' did not significantly influence PEP outcome (OR=0.45, p = 0.15). Another test used to identify factors affecting the timeliness of PEP administration was the Cox proportional hazard model. The time taken to administer PEP and "circumstances" (HR=1.13, 95%CI (0.85, 1.94), p = 0.756) and "where injury occurred" (HR=1.4, 95%CI (0.85, 1.65), p = 0.061) showed no evidence of violating the proportional hazards (PH) assumption. There was no statistical significance found with these factors and the timeliness of PEP administration. None of the participants had seroconversion with regard to HIV after the six-month period after exposure.

## Discussion

Demographics

Similar to other studies, the majority of individuals involved in NSIs in our study were women, 81.4% (n=180). This is similar to a study published in 2021 by Nagassar et al. in a hospital also located in Trinidad and Tobago, on the Eastern part of the island of Trinidad, where 76.5% (n=109) out of the 141 participants were female [5]. This may be a result of women making up the majority of the healthcare workforce. However, a study from Pakistan (2022) found a similar trend despite using equal sample groups for the study. A survey found that an equal number of men and women were involved in the study (120 for each group), and it found that women were at a higher risk of NSIs (75%) compared to men (52.5%) as a lifetime prevalence among HCWs [9].

Place where the injury occurred and where reported

The majority of injuries fall under the "other" category, 40.27% (n=89), followed by the internal medicine ward, 39.82% (n=88). The former category signifies that it did not occur on a medical ward, surgical unit, or another unit. Therefore, this includes injuries that occurred in transit or between wards and disposal. It reflects the importance of safety precautions not only in the wards but also during disposal. This emphasises the importance of strict protocols to allow for safe preparation and disposal in the ward, unit, or accident and emergency, as well as safe and consistent packaging to help workers who transport and then dispose of needles in an area in areas outside the original clinical setting. This is supported by a Nigerian multi-centre study from 2014 that emphasised that every step of handling needles, from the clinical setting to the ancillary staff and disposal, is a target point for NSIs [10]. A Serbian study with 5,247 participants (Mandić et al.) also found that the "other" category of staff, which included cleaners, was most predisposed to sharps injuries, similar to our study [11]. Although most were first reported to IPC (46.61%; n=103), a significant number were reported to the accident and emergency department (42.08%; n=03). This shows that there is a need for sensitisation and to streamline IPC practices and protocols at SFGH, especially among staff. This finding is in keeping with that of Nagassar et al. (2021), which took place in another hospital setting on the island of Trinidad. This study found that an even greater proportion of the population (n=123; 89.1%) first reported to the accident and emergency department between the period of 2007 and 2017 at the Sangre Grande Hospital [5]. This reinforces the need for national sensitisation in IPC protocols across the twin-island state of Trinidad and Tobago.

Mechanism of injury: circumstance and location on the body

A national study performed by Chadee et al. (1999) found that most inoculations occurred with NSIs nationwide, and most took place during a procedure, 58.2% (n=231) [2]. This is similar to our finding, where the main circumstance was found to be while performing a procedure, 37.11% (n=82). This varies from the findings of the study published in 2021 by Nagassar et al., where the most common mechanism was disposing of needles, accounting for 12.3% (n=17), followed by recapping of needles [5]. This variation may be a result of the categorical options used in the study. Procedures were separated into "doing an IV," “doing a blood gas", "diascan procedure," "administering an injection," and "suturing." Therefore, the sum of these would be equivalent to the major circumstance or mechanism. However, discarding and removing or recapping needles were responsible for >10% each, which is similar to the findings documented by Nagassar et al. [5]. This re-emphasises the need for IPC sensitisation.

Reporting, administration, and the type of PEP given

Making an official report to the IPC department was found to have a statistically significant association with the administration of PEP (p-value = 0.00086) (see Table 4). This is consistent with other studies, including a study from Pakistan published in 2023. This study found that 29.8% (n=68) of the HCWs experienced NSI, but only 5.7% (n=13) actually reported the injury [12]. This reflects the lack of reporting that can lead to failure to obtain PEP and follow the IPC protocol for the respective establishment. The majority of the patients received Combivir (lamivudine and zidovudine) and Kaletra (lopinavir and ritonavir), which are found to be better tolerated than other options and considered appropriate PEP, especially at the time of the study [13]. None of the participants had seroconversion after six months. The risk of seroconversion from a single sharp exposure (e.g., NSI) is approximately 1 in 333 (~ 0.3%) [14]. However, with the use of PEP, this risk decreases by 81% once given within 72 hours [15,16]. There are many variables that change these figures based on details of the situation. Considering that PEP would have been given to the majority of the participants, 94.1% (n=208) (see Table 4), their risk of seroconversion would have decreased significantly. Hence, it contributed to none of our participants having seroconversion.

Glove-wearing at the time of injury and subsequent blood tests

A notable finding is that in 80.53% (n=178) of NSI cases, it was not documented whether gloves were worn at the time of injury. This absence of documentation raises concerns about both compliance and record‑keeping. Literature consistently emphasises that gloves serve as a basic protective barrier, reducing the likelihood of direct contact with blood and bodily fluids. Although gloves do not prevent the mechanical penetration of a needle, studies suggest they may reduce the volume of fluid transferred during an injury, thereby lowering exposure risk, as the volume of blood/body fluid is also important [17].

The relevance of glove use becomes even more apparent given that most injuries occurred during procedures. Procedural NSIs typically involve close hand‑to‑needle proximity, and gloves can provide an additional layer of protection in these high‑risk moments. The lack of documentation in this study suggests an opportunity for strengthening institutional protocols and improving surveillance accuracy.

Regarding post‑exposure testing, 59.28% (n=131) of exposed individuals underwent laboratory investigations such as LFT, RFT, and CBC. These tests are commonly used to establish baseline health status following occupational exposure. An additional 22.62% (n=50) had other laboratory tests documented, including those for HIV, hepatitis B, hepatitis C, and VDRL. While these findings indicate that a proportion of exposed workers received broader evaluation, the variability in testing reflects inconsistencies in institutional practice.

Literature frequently highlights the importance of timely and consistent post‑exposure evaluation to support appropriate follow‑up and institutional monitoring [18]. Incomplete or inconsistent testing can hinder the ability to track outcomes, identify gaps, and ensure that exposed workers receive appropriate support.

In this study, variability in reporting suggests that institutional systems may not be sufficiently user‑friendly or that staff may not fully understand the importance of timely reporting. Incomplete reporting limits the ability to identify trends and implement targeted interventions.

Variables and their correlation to the time taken for PEP administration

The Kaplan-Meier survival curve by "where the injury occurred" indicated a statistically significant association with the time for PEP to be given (see Figure 2). It was found that incidents occurring in medical wards were dealt with most promptly. This resulted in these individuals having a greater chance of receiving the PEP within the 72-hour period. There are several factors that may contribute to this. The IPC doctor or infectious disease physician would likely be a consultant on these medical wards (internal medicine), and protocols would be followed closely under their direct supervision. In addition to the large proportion of injuries occurring in these wards, the process of managing the situation is also better understood. Research is scarce when it comes to a particular ward or department influencing the timeframe before obtaining PEP. However, a scoping review reflected that there are several variations and factors influencing HCWs' access to PEP. These include awareness of protocols, beliefs, and knowledge of efficacy of PEP, the stigma at the workplace with the use of PEP, and knowledge of available services [18]. In a Nigerian study, less than half (44.9%) of the HCWs at the hospital were aware of the protocol, which reflects poor overall knowledge, which may be the situation in our study [19]. Similarly, in a study performed in Ethiopia, only 40.5% of the HCW were aware that PEP was available 24 hours a day, and similarly, almost 60% were unaware that the services existed [20].

Recommendations and steps moving forward

Preventing needle‑stick injuries requires a multifaceted approach that integrates safer devices, strong institutional policies, continuous training, and a culture of safety. This study highlights the persistent risk of NSIs among HCWs in tertiary care settings and underscores the need for a comprehensive, system‑wide approach to prevention.

Thus, recommendations to strengthen safety practices, reduce occupational exposure, and enhance institutional preparedness include the following.

Strengthening training, supervision, and competency: Considering our statistically significant findings regarding the likelihood of receiving PEP once an official IPC report is made, competency in safe injection practices and sharps handling is essential for preventing NSIs. This reiterates the need for continuous, comprehensive training, reinforcing locally guided IPC practices. Institutions should implement supportive supervision. Frequent surveillance and competency assessments to identify areas requiring improvement in sharps handling would help ensure safe injection techniques. Implementing regular in‑service training programmes to update staff on evolving safety standards, device innovations, and exposure‑prevention strategies establishes continuous education that fosters a workforce that is confident, skilled, and safety‑conscious. This includes bringing National Infection Prevention and Control Policies and Guideline Manuals to the attention of both clinical and non-clinical staff to improve compliance [3,4]. Enhancing occupational exposure preparedness: A unique finding of this study was the more timely first dose of PEP when the NSI occurred in specific wards of the hospital, e.g., the medical ward (internal medicine). This shows that a similar protocol to get the NSI individual to IPC and obtain PEP should be replicated in other departments of the hospital. Preparedness for accidental exposure is essential for reducing anxiety, minimising harm, and ensuring timely support to affected staff. Institutions should ensure all HCWs are familiar with PEP protocols and understand the immediate steps required following an incident. Guidance on reporting pathways, available support, and infection‑prevention measures ensures that exposures are managed efficiently and safely. A consistent institutional response creates systems that ensure timely follow‑up, documentation, and coordinated management of exposed individuals. Institutionalising a clear PEP protocol: A clear, universally applied, standardised PEP protocol should be available in all healthcare facilities. In this way, no matter where the NSI occurs, there is a seamless process to report to IPC and obtain PEP as recommended. Rapid access to support pathways for HBV, HCV, and HIV exposure, including prompt reporting, assistance, and infection‑prevention measures, should be readily accessible in all clinical areas. Protocols, practices, and accessibility to medication may vary within a hospital depending on the location or department, indicating the need for expert opinion when in doubt. Additionally, a reliable reporting and tracking system that supports efficient management of occupational exposures and follow‑up care should be implemented. It also guides the IPC team to focus on specific departments or areas in the hospital to prevent this variation in accessing PEP, as well as align immunisation practices and information on immunoglobulin use with national guidelines to ensure consistent, evidence‑based responses. Ensuring safe sharps handling and disposal: Proper sharps disposal remains one of the most effective strategies for preventing NSIs. Healthcare facilities should use appropriate devices such as needles and syringes, evacuated needle‑and‑tube holders, or winged butterfly sets according to procedural requirements. Dispose of all sharps immediately after use in puncture‑proof, leak‑proof, enclosed sharps containers located at the point of care. To reduce the risk of injuries associated with improper disposal or handling of used sharps, ensure that sharps containers are not overfilled and are replaced promptly. This can be adapted to already established national policies and guidelines [3,4]. Strengthening product safety and quality: Improving the safety of medical devices is central to reducing NSIs. Healthcare institutions should work closely with procurement agencies to ensure continuous improvement in product selection and supply chain reliability and to prioritise the procurement of devices that are both high‑quality and cost‑effective, ensuring that safety is not compromised by budget constraints. Collaboration with manufacturers on clearly defined clinical needs and safety expectations reduces the likelihood of device‑related injuries. Structured evaluation of improved device designs ensures compliance with quality and usability standards. Routine surveillance to identify product defects, device‑related adverse events, and recurring patterns of equipment failure supports the adoption of safer technologies.

Limitations and assumptions

This was a retrospective study and involved a review of processes that had already been completed. Although this was a multi-year study that involved real-world data analysis and assessing comprehensive outcomes, there were several limitations to note. The sample size of 296 was not met, but this was a result of the number of cases reported within the timeframe of 2011 to 2016. What is documented is assumed to be the most accurate and correct data available to the researchers. The retrospective review is limited by missing data and failure to document, compared to a prospective study. It is assumed that all those who required PEP met the criteria to obtain this, and also that the local protocols were followed. However, the recommendations presented emphasise the need for safer medical devices, strengthened occupational exposure preparedness, improved training and supervision, and reliable reporting systems. Implementing these measures can significantly reduce the incidence of NSIs, enhance staff confidence, and promote a safer working environment. Furthermore, aligning institutional practices with national guidelines ensures consistency, accountability, and improved outcomes for exposed individuals.

## Conclusions

This retrospective study provides valuable insight into the patterns, contributing factors, and institutional gaps associated with NSIs. The findings highlight that NSIs frequently occur during the discarding, removing, or recapping of needles, underscoring the importance of safe‑practice adherence, adequate training, and the consistent use of protective equipment. The most common area of obtaining needle stick injury was “Other” areas outside the wards and units, which re-emphasises the proper care beyond the clinical area, regarding disposal of needles after their use. There is room for improvement in reporting and documenting NSI, so that there is no delay in obtaining PEP as deemed necessary. This needs to be standardized throughout the institution to prevent variations in reporting and obtaining PEP. This is reflected in the finding that those who made an official IPC report were more likely to obtain PEP. Ultimately, a culture of safety, adequate training, and support at tertiary care hospitals can reduce occupational risks.

## Appendices

1. Age

2. Gender:

Male; Female

3. Speciality:

A&E; Internal Med; O&G Gen Surgery; Pediatrics; Other - please specify

4. Status:

Clinician; Student/trainee; Other

5. Method of needle stick, e.g., recapping needle

6. Circumstances under which the event occurred, e.g., performing a procedure, recapping/removing needle, other

7. Site of injury

8. Time between the event and the reporting of the incident

9. Where did the injury occur:

Ward A&E Surgical/Uro/Paed/Ortho/ENT; Other unit - please specify

10. Physician review:

Yes; No

11. Where first reported:

A&E; IPC unit; Health centre; Other

12. Wearing gloves:

Yes; No

13. Blood tests performed:

CBC/LFT/RFT/HIV/HepB/HepC +/- VDRL

14. PEP:

Yes; No. If Yes, which medications, how long after injury?

15. Official report to IPC unit filed:

Yes; No

16. Seroconversion of health care workers after 6 months

17. Other reported occupational exposure

## References

[1] Prüss-Üstün A, Rapiti E, Hutin Y (2023). Sharps Injuries: Global Burden of Disease From Sharps Injuries to Health-Care Workers. https://www.who.int/publications/i/item/9241562463.

[2] Chadee DD, Edwards JR, Sharma K, Shivnath B (1999). Inoculation injuries in hospitals in Trinidad and Tobago. West Indian Med J.

[3] (2026). Ministry of Health, Government of Trinidad and Tobago. Occupational health and dentistry: guidelines for infection prevention and control. https://health.gov.tt/sites/default/files/2022-03/PAHO%20MoH%20Manual_OCCUPATIONAL%20HEALTH%20AND%20DENTISTRY.pdf.

[4] (2026). Ministry of Health, Government of Trinidad and Tobago. Infection prevention and control: policies and guidelines for health care services. https://health.gov.tt/sites/default/files/2022-03/PAHO%20MoH%20Manual_INFECTION%20PREVENTION%20AND%20CONTROL%20POLICIES%20.pdf.

[5] Nagassar RP, Daniel K, Ramkissoon S, Bridgelal-Nagassar RJ (2021). The epidemiology of sharp injuries in healthcare workers at a Trinidadian community hospital. Caribb Med J.

[6] Lwanga SK, Lemeshow S (1991). Sample Size Determination in Health Studies: A Practical Manual. https://apps.who.int/iris/handle/10665/40062.

[7] (2026). Raosoft Inc. Sample size calculator. http://web.archive.org/web/20250628045932/http://www.raosoft.com/samplesize.html.

[8] Matsubara C, Sakisaka K, Sychareun V, Phensavanh A, Ali M (2017). Prevalence and risk factors of needle stick and sharp injury among tertiary hospital workers, Vientiane, Lao PDR. J Occup Health.

[9] Altaf A, Javaid A, Shabir M (2022). Relationship of gender to prevalence, knowledge, attitude and practice among healthcare workers regarding needle-stick injuries in tertiary care hospitals. Pak J Public Health.

[10] Amira CO, Awobusuyi JO (2014). Needle-stick injury among health care workers in hemodialysis units in Nigeria: a multi-center study. Int J Occup Environ Med.

[11] Mandić B, Mandić-Rajčević S, Marković-Denić L, Bulat P (2018). Occupational exposure to blood and bodily fluids among healthcare workers in Serbian general hospitals. Arh Hig Rada Toksikol.

[12] Rahman S, Hayat S, Ahmad A, Ali NM (2023). Barriers to the adoption of Infection Prevention Control (IPC) guidelines among health care workers at Saidu Group of Teaching Hospital (SGTH), Swat - a cross sectional study. Int J Med Res Health Sci.

[13] Rabaud C, Burty C, Grandidier M (2005). Tolerability of postexposure prophylaxis with the combination of zidovudine-lamivudine and lopinavir-ritonavir for HIV infection. Clin Infect Dis.

[14] Asanati K, Majeed A, Shemtob L, Cresswell F (2022). Healthcare workers potentially exposed to HIV: an update. J R Soc Med.

[15] World Health Organization (2025). Guidelines for HIV post-exposure prophylaxis. https://www.who.int/publications/i/item/9789240095137.

[16] Cardo DM, Culver DH, Ciesielski CA (1997). A case-control study of HIV seroconversion in health care workers after percutaneous exposure. Centers for Disease Control and Prevention Needlestick Surveillance Group. N Engl J Med.

[17] (2010). WHO Guidelines on Drawing Blood: Best Practices in Phlebotomy. Geneva: World Health Organization; 2010. 8, Implementing best phlebotomy practices. Available from. WHO Guidelines on Drawing Blood: Best Practices in Phlebotomy.

[18] Auerbach JD, Malone S, Forsyth AD (2024). Occupational post-exposure prophylaxis among healthcare workers: a scoping review of factors affecting optimal utilization. J Int AIDS Soc.

[19] Oche OM, Umar AS, Gana GJ, Okafoagu NC, Oladigbolu RA (2018). Determinants of appropriate knowledge on human immunodeficiency virus postexposure prophylaxis among professional health-care workers in Sokoto, Nigeria. J Family Med Prim Care.

[20] Degavi G, Adola SG, Panari H, Pawar S, Dereso CW (2020). Prevalence of occupational exposure to HIV and utilization of HIV post-exposure prophylaxis among health staff at Bule Hora General Hospital, Bule Hora, Ethiopia. Pan Afr Med J.

